# Associations Between Antihypertensive Medications and Severe Hyponatremia: A Swedish Population–Based Case–Control Study

**DOI:** 10.1210/clinem/dgaa194

**Published:** 2020-04-14

**Authors:** Henrik Falhammar, Jakob Skov, Jan Calissendorff, David Nathanson, Jonatan D Lindh, Buster Mannheimer

**Affiliations:** 1 Department of Molecular Medicine and Surgery, Karolinska Institutet, Stockholm, Sweden; 2 Department of Endocrinology, Metabolism and Diabetes, Karolinska University Hospital, Stockholm, Sweden; 3 Department of Medicine, Karlstad Central Hospital, Karlstad, Sweden; 4 Department of Medicine, Karolinska University Hospital Huddinge, Karolinska Institutet, Stockholm, Sweden; 5 Department of Clinical Science and Education, Södersjukhuset, Karolinska Institutet, Stockholm, Sweden; 6 Department of Laboratory Medicine, Division of Clinical Pharmacology, Karolinska University Hospital Huddinge, Karolinska Institutet, Stockholm, Sweden

**Keywords:** calcium channel blockers, beta-receptor blockers, angiotensin converting enzyme inhibitors, angiotensin II receptor blockers, hospitalization, hyponatremia, SIADH, adverse reaction

## Abstract

**Background:**

Calcium channel blockers (CCBs), beta-receptor blockers (BBs), angiotensin-converting enzyme inhibitors (ACEIs), and angiotensin II receptor blockers (ARBs) have occasionally been reported to cause severe hyponatremia. The aim was to explore the association between CCBs, BBs, ACEIs, and ARBs and hospitalization due to hyponatremia.

**Methods:**

Patients hospitalized with a principal diagnosis of hyponatremia (*n* = 11 213) were compared with matched controls (*n* = 44 801). Linkage of national population-based registers was used to acquire data. Multivariable logistic regression adjusting for co-medications, diseases, previous hospitalizations, and socioeconomic factors was used to explore the association between hospitalization for severe hyponatremia and the use of different CCBs, BBs, ACEIs, and ARBs. Furthermore, newly initiated (≤90 days) and ongoing use were examined separately.

**Results:**

Adjusted odds ratios (aORs) (95% confidence interval) for the investigated 4 drug classes ranged from 0.86 (0.81-0.92) for CCBs to 1.15 (1.07-1.23) for ARBs. For newly initiated drugs, aORs spanned from 1.64 (1.35-1.98) for CCBs to 2.24 (1.87-2.68) for ACEIs. In contrast, the corresponding associations for ongoing therapy were not elevated, ranging from 0.81 (0.75-0.86) for CCBs to 1.08 (1.00-1.16) for ARBs. In the CCBs subgroups, aOR for newly initiated vascular CCBs was 1.95 (1.62-2.34) whereas aOR for ongoing treatment was 0.82 (0.77-0.88).

**Conclusions:**

For newly initiated CCBs, BBs, ACEIs, and ARBs, the risk of hospitalization due to hyponatremia was moderately elevated. In contrast, there was no evidence that ongoing treatment with investigated antihypertensive drugs increased the risk for hospitalization due to hyponatremia.

In hospitalized patients, electrolyte disturbances are frequent (1,2), with up to 30% being affected by hyponatremia. Hyponatremia can be a life-threatening condition with seizures, coma, and death (due to brain edema) but more often displays mild, nonspecific symptoms (eg, lethargy, gait abnormality, confusion) (3-7). One of the most frequent causes of hyponatremia is drugs, primarily thiazide diuretics, antidepressants, antiepileptic drugs and antipsychotics, but many other drugs can also induce hyponatremia (8-16).

The 5 major drug classes in hypertension treatment are calcium channel blockers (CCBs), beta-receptor blockers (BBs), angiotensin-converting enzyme inhibitors (ACEIs), angiotensin II receptor blockers (ARBs), and thiazide diuretics (17). These drugs are used by a large proportion of the population, since the prevalence of hypertension is approximately 30% to 45% in the adults and more than 60% in individuals 60 years old or older (18). Thiazide diuretics are well-known to induce hyponatremia (6), but whether hyponatremia can be induced by the other 4 major drug classes is more controversial (8). Mostly case reports or smaller observational studies have described hyponatremia induced by CCBs (19-21), BBs (21), ACEIs (21-25), and ARBs (21,26). Due to the considerable prescription of these drugs, even a rare adverse reaction may have considerable clinical implications. Thus, the evidence of an association between antihypertensives and hyponatremia remains insufficient, and this may cause issues for the treating physician and the patient.

The primary aim of this study was to investigate the association between treatment with CCBs, BBs, ACEIs, and ARBs and hospitalization due to hyponatremia. Secondarily, we aimed to evaluate newly initiated versus ongoing use of these medications to investigate any time-dependent effects.

## Methods

This was a retrospective case–control study of the Swedish general population from October 1, 2005 to December 31, 2014. On each admission, the principal diagnosis was used due to its reflection on the main cause of hospitalization. All admissions and outpatient visits in Sweden are coded by physicians using the *International Classification of Diseases*, 10th revision (ICD10) (27). All adult persons, 18 years old or older, who had been hospitalized with a first-ever (defined as not occurring since January 1, 1997) principal ICD10 code of E87.1 (hyponatremia) or E22.2 (syndrome of inappropriate antidiuretic hormone secretion [SIADH]) in the National Patient Register (NPR; see following discussion) were classified as cases during the study period. From the Total Population Register controls, age-, sex-, and municipality-matched 4:1 per case, with no previous diagnosis of hyponatremia (since January 1, 1997) were randomly identified. A validation study of the principal diagnosis of hyponatremia, with sodium levels corrected for glucose levels, was performed in one of the larger hospitals (11). The same cohort has been used in previous studies to analyze other aspects of hyponatremia (7,11-16,28).

By using ICD10 codes, Anatomical Therapeutic Chemical (ATC) codes and parameters from the longitudinal integration database for health insurance and labor market studies (LISA)-register possible confounders for hyponatremia were identified (11). All included variables in the multivariable logistic regression analysis are depicted in the online repository (29). Exposure to CCBs, BBs, ACEIs, and ARBs was defined as documented dispensations within 90 days prior to the index date (date of hospitalization with a principal diagnosis of hyponatremia). The index date of matched controls was the hospitalization date of their case. The 90-day timeframe was chosen because virtually all drugs used in chronic conditions are dispensed every 90 days in Sweden (11). Adjustment for concurrent disorders was done since January 1, 1997 to the index date, with the exception of infectious diseases, which were adjusted for within 90 days before the index date. Only drugs being used in the cohort during the study period were included in the analysis. CCBs with mainly vascular effects were defined as vascular CCBs and CCBs with direct cardiac effects were defined as cardiac CCBs (for the individual drugs in each group; see Table 1). Newly initiated use of a drug was defined as a dispensation within 90 days prior to the index date preceded by at least 12 months without exposure to the drug. The definition of ongoing use of the drug or drug class required one or more dispensations in the period 91 to 454 days before the index date as well.

**Table 1.** Prevalence of important comorbidities and drugs among cases (hospitalized with a principal diagnosis of hyponatremia) and controls

	Cases (*N* = 14 359) *n* (%)	Controls (*N* = 57 382) *n* (%)
**Diagnosis**		
Malignancy	3826 (26.6)^***^	11 251 (19.6)
Ischemic heart disease	2808 (19.6)^***^	7880 (13.7)
Alcoholism	2285 (15.9)^***^	1028 (1.8)
Congestive heart failure	1900 (13.2)^***^	4493 (7.8)
Cerebrovascular disease	1884 (13.1)^***^	4540 (7.9)
COPD	1477 (10.3)^***^	1958 (3.4)
Hypothyroidism	1439 (10.0)^***^	2396 (4.2)
Adrenal insufficiency	586 (4.1)^***^	340 (0.6)
Renal disease	631 (4.4)^***^	1098 (1.9)
Liver disease	553 (3.9)^***^	417 (0.7)
Pancreatic disease	327 (2.3)^***^	513 (0.9)
Inflammatory bowel disease	285 (2.0)^***^	533 (0.1)
**Medications**		
Antidepressants	3517 (24.5)^***^	7079 (12.3)
Antipsychotics	1008 (7.0)^***^	1373 (2.4)
Antiepileptic drugs	1355 (9.4)^***^	1366 (2.4)
Furosemide	2236 (15.6)^***^	6903 (12.0)
Thiazides	5204 (36.2)^***^	7425 (12.9)
**Proxy for frailty**		
Number of dispensed drugs 90 days prior to index date		
<4 drugs	2900 (20.2)^***^	30 480 (53.1)
4-7 drugs	4162 (29.0)^**^	15 930 (27.8)
8-12 drugs	4604 (32.1)^***^	8596 (15.0)
>12 drugs	2693 (18.8)^***^	2376 (4.1)
Number of hospitalizations ≥3 days duration	6409 (44.6)^***^	12 214 (21.3)
**Antihypertensive medication, total**		
One CCB, BB, ACEI, or ARB	3855 (26.8)^***^	12 388 (21.6)
More than 1 of CCB, BB, ACEI, and ARB	4829 (33.6)^***^	10 927 (19.0)
***Any CCB***	3237 (22.5)^***^	7780 (13.6)
*Vascular CCBs*	3078 (21.4)^***^	7186 (12.5)
Amlodipine	1539 (10.7)^***^	3279 (5.7)
Felodipine	1460 (10.2)^***^	3654 (6.4)
Nifedipine	60 (0.4)^***^	125 (0.2)
Lercanidipine	39 (0.3)^***^	66 (0.1)
*Cardiac CCBs*	178 (1.2)	610 (1.1)
Verapamil	90 (0.6)	351 (0.6)
Diltiazem	88 (0.6)^*^	260 (0.5)
***Any BB***	5256 (36.6)^***^	13 919 (24.3)
*Nonselective BBs*	436 (3.0)^***^	911 (1.6)
Propranolol	281 (2.0)^***^	475 (0.8)
Sotalol	128 (0.9)^***^	344 (0.6)
*Selective BBs*	4766 (33.2)^***^	12 809 (22.3)
Metoprolol	2876 (20.0)^***^	7915 (13.8)
Atenolol	1220 (8.5)^***^	3087 (5.4)
Bisoprolol	750 (5.2)^***^	1859 (3.2)
*ABBs*	112 (0.8)^***^	241 (0.4)
Carvedilol	108 (0.8)^***^	226 (0.4)
***Any ACEI***	3097 (21.6)^***^	7580 (13.2)
Enalapril	2422 (16.9)^***^	5619 (9.8)
Lisinopril	53 (0.4)	181 (0.3)
Ramipril	586 (4.1)^***^	1649 (2.9)
***Any ARB***	2841 (19.8)^***^	5872 (10.2)
Losartan	1300 (9.1)^***^	2789 (4.9)
Valsartan	197 (1.4)^***^	387 (0.7)
Irbesartan	221 (1.5)^***^	394 (0.7)
Candesartan	1108 (7.7)^***^	2218 (3.9)
**Antihypertensive medication, recently initiated treatment**		
One CCB, BB, ACEI, or ARB	945 (6.6)^***^	1121 (2.0)
More than 1 of CCB, BB, ACEI, and ARB	976 (6.8)^***^	723 (1.3)
***Any CCB***	888 (6.2)^***a^	548 (1.0)
*Selective CCBs with mainly vascular effects*	890 (6.2)^***^	520 (0.9)
Amlodipine	552 (3.8)^***^	323 (0.6)
Felodipine	437 (3.0)^***^	234 (0.4)
Nifedipine	13 (0.09)^***^	8 (0.01)
Lercanidipine	12 (0.08)^***^	9 (0.02)
*Selective CCBs with direct cardiac effects*	22 (0.2)^**^	38 (0.07)
Verapamil	11 (0.08)	22 (0.04)
Diltiazem	11 (0.08)^*^	17 (0.03)
***Any BB***	599 (4.2)^***^	737 (1.3)
*Nonselective BBs*	62 (0.4)^***^	86 (0.1)
Propranolol	47 (0.3)^***^	59 (0.1)
Sotalol	14 (0.1)^**^	21 (0.04)
*Selective BBs*	597 (4.2)^***^	679 (1.2)
Metoprolol	449 (3.1)^***^	445 (0.8)
Atenolol	104 (0.7)^***^	134 (0.2)
Bisoprolol	165 (1.1)^***^	185 (0.3)
*ABBs*	18 (0.1)^***^	13 (0.02)
Carvedilol	17 (0.1)^***^	12 (0.02)
***Any ACEI***	605 (4.2)^***^	546 (1.0)
Enalapril	497 (3.5)^***^	447 (0.8)
Lisinopril	2 (0.003)	3 (0.005)
Ramipril	123 (0.9)^***^	109 (0.2)
***Any ARB***	371 (2.5)^***^	346 (0.6)
Losartan	228 (1.6)^***^	220 (0.4)
Valsartan	26 (0.2)^***^	13 (0.02)
Irbesartan	14 (0.1)^**^	20 (0.03)
Candesartan	161 (1.1)	129 (0.2)

Abbreviations: ABBs, alpha- and beta-blockers; ACEIs, angiotensin converting enzyme inhibitors; ARBs, angiotensin II receptor blockers; BBs, beta-receptor blockers; CCBs, calcium channel blockers; COPD, chronic obstructive pulmonary disease.

*P < 0.05.

^**^ *P* < 0.01.

^***^ *P* < 0.001 compared to controls.

^a^Patients switching from a cardiac CCB to a vascular CCB were counted as newly initiated on a vascular CCB, but not as newly initiated on any CCB. This explains why the number of patients newly initiated on vascular CCBs exceeds the number of patients newly initiated on any CCB.

To investigate the robustness of our results a sensitivity analysis was performed on a healthier subpopulation without diagnostic records of heart or kidney failure, ischemic heart disease or filled prescriptions of thiazide diuretics within 90 days of baseline.

The unique Swedish personal identification number made linkage between the population-based registers possible. The NPR, the Swedish Prescribed Drug Register and the LISA register were all linked (27,30,31). The NPR contains all the ICD10 codes (since 1997), while the Swedish Prescribed Drug Register contains all prescriptions dispensed in Sweden (since July 1, 2005). To adjust for socioeconomic variables, the LISA register was utilized.

The Regional Ethical Review Board in Stockholm approved the study, and due to its retrospective epidemiological nature, no informed consent was required.

### Statistical analysis

The associations between hospitalization with a principal diagnosis of hyponatremia and exposure to a CCB, BB, ACEI, and/or ARB were analyzed by means of univariable and multivariable logistic regression. For each variable included in the multivariable regression, absence of exposure was used as reference (eg, an adjusted odds ratio [aOR] of 1.38 for propranolol indicates an increased risk in propranolol users since no propranolol exposure was used as reference). In addition, the specific effects of newly initiated and ongoing treatment were investigated in multivariable models with exposure to each antihypertensive drug (or class of drugs) classified as ongoing, newly initiated, or absent. In these models, adjustment for other antihypertensives included the timing of these drugs (ongoing vs newly initiated treatment). Moreover, the distribution of model covariates were compared between cases/controls with any newly initiated therapy. The associations between the individual drugs and hospitalization due to hyponatremia in cases and controls were reported as unadjusted and adjusted odds ratios, with 95% confidence intervals (95% CI). In a separate adjusted analysis, drug exposure was divided into newly initiated (≤90 days) and ongoing (>90 days) treatment, encoded as separate binary variables. *P*-values < 0.05 were considered statistically significant. For all analyses, R version 3.6.1 was used (32).

## Results

A principal diagnosis of hyponatremia was found on the admission in 11 213 individuals, 18 years or older, over the 9-year study period. In addition, 44 801 matched controls were identified. The majority were females (65%) and elderly (median age 76 years [range 18-103]). Table 1 depicts medical conditions, other medications, and the use of CCBs, BBs, ACEIs, and ARBs at baseline (index date). Besides hyponatremia, the most frequently recurring medical diagnoses were malignancies, ischemic heart disease, and alcoholism. The most common types of antihypertensive drugs were BBs, ACEIs, and CCBs, dispensed to 27%, 16%, and 16% of the total population, respectively.

The association between exposure to CCBs, BBs, ACEIs, and ARBs and hyponatremia hospitalization is presented in the online repository (29). The associations for all investigated groups were elevated with unadjusted (95% CI) odds ratios ranging from 1.53 (1.45-1.61) for BBs to 2.11 (2.00-2.23) for ARBs. After adjustment for potential confounders, the associations decreased. aORs (95% CI) spanned from 0.86 (0.81-0.92) for CCBs to 1.15 (1.07-1.23) for ARBs. For individual drugs, aORs (95% CI) ranged from 0.57 (0.46-0.76) for verapamil to 1.44 (0.94-2.18) for nifedipine.

**Figure 1.**
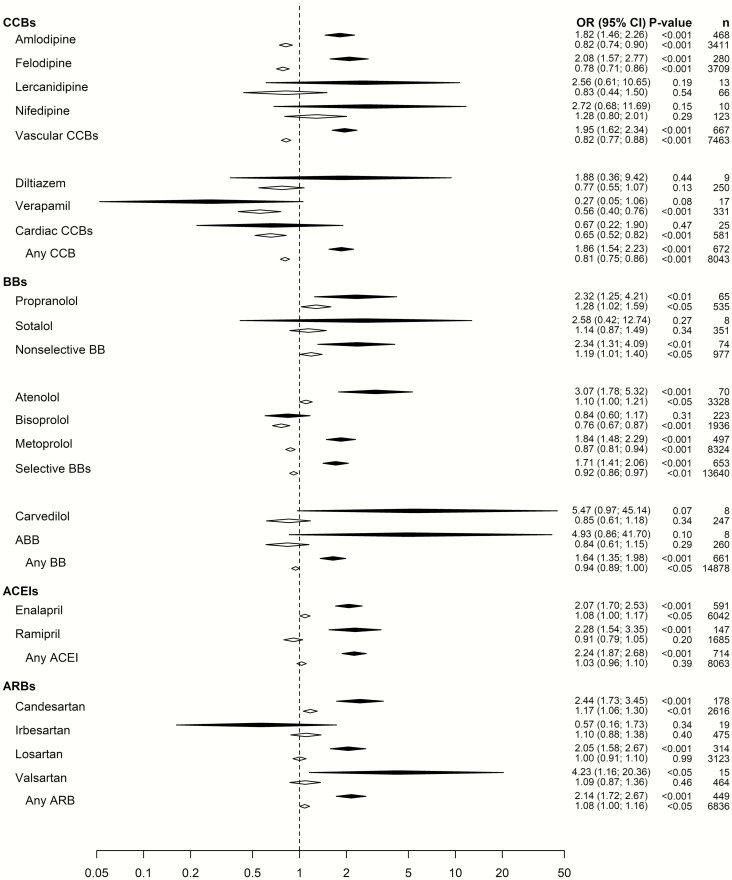
The odds ratios (95% CI) for hospitalization due to severe hyponatremia in patients with ongoing (white) or newly initiated (black) calcium channel blockers, beta-receptor blockers, angiotensin converting enzyme inhibitors, or angiotensin II receptor blockers. All odds ratios have been adjusted for the confounding factors as detailed in the online repository (29).

In Fig. 1, aORs for newly initiated use of a drug versus ongoing therapy is presented. For newly initiated use of a drug class, aORs (95% CI) ranged from 1.64 (1.35-1.98) for BBs to 2.24 (1.87-2.68) for ACEIs. In contrast, the corresponding associations for severe hyponatremia for ongoing therapy were not elevated ranging from 0.81 (0.75-0.86) for CCBs to 1.08 (1.00-1.16) for ARBs. In the CCBs subgroups, the aOR for newly initiated vascular CCBs was 1.95 (1.62-2.34) whereas the aOR for ongoing treatment was 0.82 (0.77-0.88). For cardiac CCBs, corresponding aORs were 0.67 (0.22-1.90) and 0.65 (0.52-0.82), respectively.

**Figure 2.**
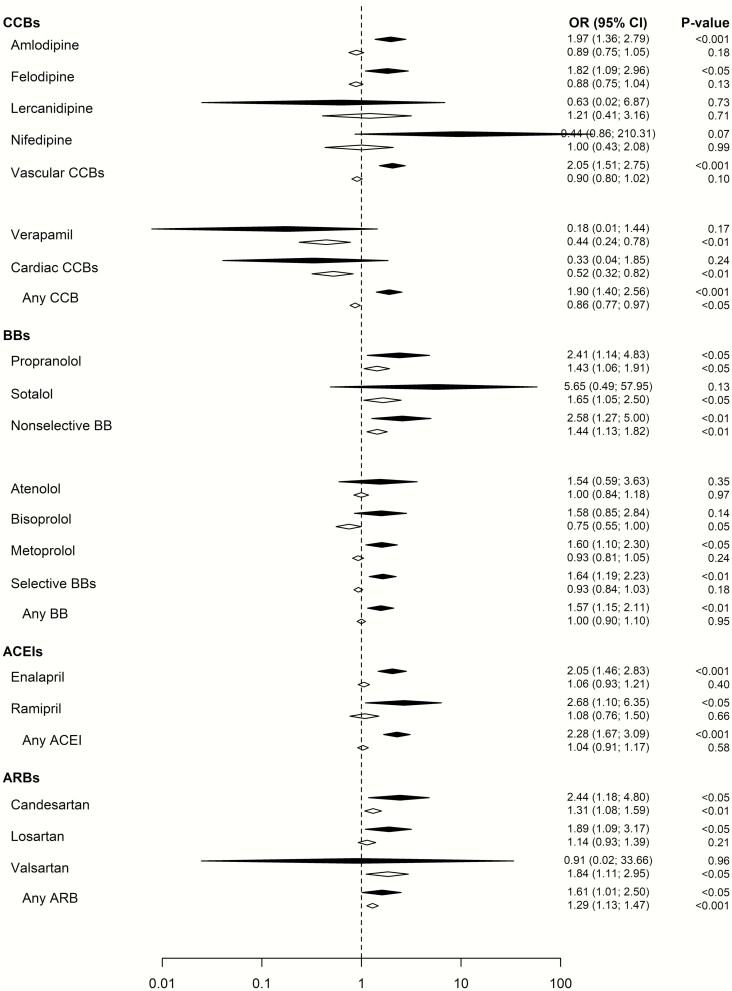
Sensitivity analysis performed on a healthier subpopulation without diagnostic records of heart or kidney failure, ischemic heart disease, or filled prescriptions of thiazide diuretics. The odds ratio (95% CI) is provided for hospitalization due to severe hyponatremia in patients with ongoing (white) or newly initiated (black) calcium channel blockers, beta-receptor blockers, angiotensin converting enzyme inhibitors, or angiotensin II receptor blockers. All odds ratios have been adjusted for the confounding factors as detailed in the online repository (29).

To investigate the robustness of our results a sensitivity analysis was performed on a healthier subpopulation without diagnostic records of heart or kidney failure, ischemic heart disease, or filled prescriptions of thiazide diuretics. The results remained essentially similar (Fig. 2).

Comorbidities, co-medication and other risk factors for hyponatremia were all more common among hyponatremic cases newly initiated on antihypertensives than among controls newly initiated on the same antihypertensives (Table 2).

**Table 2.** Medical characteristics of subjects with newly initiated use of calcium channel blockers, beta-receptor blockers, angiotensin converting enzyme inhibitors and angiotensin ii receptor blockers in subjects hospitalized due to hyponatremia (cases) versus subjects with newly initiated use with the same drugs but not hospitalized due to hyponatremia (controls)

	Cases with newly initiated therapy (*N* = 2037) *n* (%)	Controls with newly initiated therapy (*N* = 1886) *n* (%)	Newly initiated therapy in cases vs. controls Unadjusted OR (95% CI)
**Diagnosis**			
Malignancy	489 (24.0)	395 (20.9)	1.19 (1.03-1.39)^*^
Ischemic heart disease	383 (18.8)	409 (21.7)	0.84 (0.72-0.98)^*^
Alcoholism	211 (10.4)	28 (1.5)	7.67 (5.14-11.43)^***^
Congestive heart failure	248 (12.2)	236 (12.5)	0.97 (0.80-1.17)
Cerebrovascular disease	263 (12.9)	188 (10.0)	1.34 (1.10-1.63)^**^
COPD	186 (9.1)	89 (4.7)	2.03 (1.56-2.63)^***^
Hypothyroidism	210 (10.3)	88 (4.7)	2.35 (1.82-3.04)^***^
Adrenal insufficiency	76 (3.7)	19 (1.0)	3.81 (2.29-6.32)^***^
Renal disease	69 (3.4)	60 (3.2)	1.07 (0.75-1.52)
Liver disease	38 (1.9)	14 (0.7)	2.54 (1.37-4.71)^***^
Pancreatic disease	29 (1.4)	22 (1.1)	1.22 (0.70-2.14)
Inflammatory bowel disease	26 (1.3)	14 (0.7)	1.73 (0.90-3.32)
**Medications**			
Antidepressants	473 (23.2)	242 (12.8)	2.05 (1.73-2.43)^***^
Antipsychotics	84 (4.1)	39 (2.1)	2.04 (1.39-2.99)^***^
Antiepileptic drugs	137 (6.7)	40 (2.1)	3.33 (2.33-4.76)^***^
Furosemide	344 (16.9)	402 (21.3)	0.75 (0.64-0.88)^***^
Thiazides	1103 (54.1)	455 (24.1)	3.71 (3.24-4.26)^***^
**Proxy for frailty** Number of dispensed drugs 90 days prior to index date			
<4 drugs	157 (7.7)	459 (24.3)	0.26 (0.21-0.32)^***^
4-7 drugs	637 (31.3)	788 (41.8)	0.63 (0.56-0.72)^***^
8-12 drugs	779 (38.2)	469 (24.9)	1.87 (1.63-2.15)^***^
>12 drugs	464 (22.8)	170 (9.0)	2.98 (2.47-3.60)^***^
Number of hospitalizations ≥3 days duration	815 (40.0)	660 (35.0)	1.24 (1.09-1.41)^*^

Newly initiated use was defined as a dispersion of a calcium channel blocker, a beta-receptor blocker, an angiotensin converting enzyme inhibitor, and an angiotensin II receptor blocker within 90 days prior to the index date preceded by at least 12 months without exposure to the same drug.

Abbreviations: 95% CI, 95% confidence interval; COPD, chronic obstructive pulmonary disease; OR, odds ratio.

*P < 0.05.

^**^ *P* < 0.01.

^***^ *P* < 0.001 comparing newly initiated therapy in cases vs controls.

## Discussion

This is the first nationwide population-based case-control study reporting on CCBs, BBs, ACEIs, and ARBs and hospitalization due to severe hyponatremia. The risk associated with newly initiated CCBs, BBs, ACEIs, and ARBs was moderately elevated. In contrast, there was no evidence suggesting that ongoing treatment with any of the investigated antihypertensive drug classes increased the risk for hospitalization due to hyponatremia.

Previous evidence of CCB-induced severe hyponatremia is restricted to occasional case reports, mainly concerning the vascular CCB amlodipine (20,21). Increased risk of moderate hyponatremia in psychiatric patients using CCBs has also been reported (19), but considering the widespread use of CCBs and the scarcity of data linking them to hyponatremia, they are generally considered safe in this context. In fact, treatment recommendations sometimes propose switching hyponatremic patients on other antihypertensive agents to CCBs (33). The finding of a 2-fold increase in risk associated with newly initiated CCBs is therefore unexpected.

To test the robustness of these results we performed a sensitivity analysis on a healthier subpopulation. By excluding thiazide use in the sensitivity analysis, we addressed the possibility that some of the associations were explained by a switch from thiazides to vascular CCBs, being undetected by the primary main analysis. To address the possibility of an imbalance of the therapeutic indications for respective drug being associated with differences in morbidity between the investigated groups, we also excluded individuals with heart or kidney failure or ischemic heart disease in the sensitivity analysis. However, as these results remained essentially similar, this sensitivity analysis supports our primary findings. Moreover, as seen in Table 2, cases newly initiated on CCBs, BBs, ACEIs, or ARBs had more comorbidities and co-medications compared to controls newly initiated on the same drugs. Importantly, the very purpose of the regression model including all these factors was to control for this imbalance producing aORs that reflect the independent risk attributed to the investigated antihypertensive drugs. However, the results in Table 2 is valuable, indicating which factors that for the individual patient may be of importance for the development of hyponatremia.

The antihypertensive effect of CCBs is mediated through peripheral vasodilation. However, CCBs also have natriuretic properties, possibly through direct effects on renal proximal tubular sodium reabsorption (34,35). The result suggests that the increased risk is restricted to vascular CCBs. However, for cardiac CCBs, the analysis was based on only 25 individuals resulting in a wide confidence interval, weakening the conclusion in this regard, and a class effect for all CCBs cannot be ruled out.

Occasional studies have linked BBs with severe hyponatremia, mainly atenolol but also bisoprolol and propranolol (21). We found that propranolol and atenolol were associated with an increased risk of severe hyponatremia regardless of treatment duration, while the other BBs only correlated with hyponatremia when newly initiated. The potential mechanism of action is unclear, but BBs indirectly inhibit angiotensin II via inhibition of renin secretion; this, in turn, may lead to hyponatremia (see the following discussion about ARBs).

There are several reports about ACEI-induced severe hyponatremia (21-25). In the majority of cases enalapril is the culprit (21,23), but lisinopril (21,23,24), ramipril (21,25), and other ACEIs have also been implicated (21,23). The mechanism by which ACEIs may induce severe hyponatremia is unclear. SIADH, has been the suggested as a plausible mechanism (23,24), but it may be multifactorial (25). In low to moderate doses, ACEIs may inhibit the conversion of angiotensin I to angiotensin II peripherally but not centrally in the brain (36). Elevated concentrations of angiotensin I centrally are converted to angiotensin II, which stimulates thirst and arginine vasopressin (AVP) secretion. Animal studies have shown a markedly increased AVP secretion after intracerebroventricular injection of angiotensin II (37), supporting this mechanism.

ARBs were developed as a more specific drug blocking the angiotensin II receptor. ARBs have been associated with severe hyponatremia in some cases (21,26). It has been speculated that blocking the angiotensin I receptor leads to an angiotensin II–mediated decrease in renal tubular sodium reabsorption resulting in hyponatremia (26). However, most cases of ARB-induced severe hyponatremia seemed to have occurred in patients on ARBs in combination with a thiazide diuretic (38). For angiotensin-converting enzyme inhibitors, SIADH secondary to brain angiotensin II has been suggested as a plausible mechanism leading to hyponatremia (see previous discussion). However, nonosmotic AVP release caused by hemodynamic effects from antihypertensive treatment, regardless of drug class, is perhaps a more likely explanation considering the uniform effects observed here.

Interestingly, a temporal association between initiation of CCBs, BBs, ACEIs, and ARBs and hospitalization due to hyponatremia was evident; that is, the risk of severe hyponatremia appeared to be higher for drugs newly commenced compared to ongoing treatment. The risk for severe hyponatremia for ongoing therapy with CCBs and BBs was even lower than for controls. We have previously demonstrated similar patterns with reduced risk of severe hyponatremia during ongoing therapy with antidepressants (11), antiepileptic drugs (12), proton pump inhibitors (13), and mild opioids (15). We hypothesize that individuals predisposed for drug-related severe hyponatremia ceased the treatment shortly after initiation, leaving patients that were less susceptible on continued medication.

There are several strengths and limitations to the present study. The major strength is the inclusion of all patients admitted with a principal diagnosis of severe hyponatremia in the entire country during almost a decade. Thanks to the large size of this study, comparisons between a wide range of individual CCBs, BBs, ACEIs, and ARBs could be made, albeit with uncertain estimates for drugs rarely prescribed. Restricting analysis to patients hospitalized with a main diagnosis of hyponatremia, in contrast to studies including individuals with hyponatremia as a secondary diagnosis, hyponatremia diagnosed in secondary care (9), or patients with a mild to moderate hyponatremia regardless of symptoms (10), where the clinical relevance of hyponatremia can be questioned, is an added strength. One major limitation was that the exact sodium levels were not available. To make sure that our study design only included clinically relevant hyponatremia, we have previously performed a validation study on patients admitted in one of the major Swedish hospitals (11). We found that those admitted with a principal diagnosis of hyponatremia 89% had been hospitalized predominantly due to symptoms of hyponatremia with a mean plasma sodium level of 121 mmol/L. Moreover, 77% had profound hyponatremia (11) (ie, a level less than <125 mmol/L) (6). Furthermore, despite adjusting for a broad range of comorbidities and medications, we cannot exclude residual confounding that may have biased the results. Patients with hyponatremia often suffer from multiple comorbidities, something we did not take into account during matching of cases and controls. We did, however, adjust for multiple comorbidities and frailty at a later stage. Another statistical approach to address the methodological limitation of discrepant groups would have been to use propensity-score matching. In a cohort study with a large number of individuals that have not suffered from the investigated outcome, propensity-score matching provides satisfying possibilities to correct for these imbalances. However, the current study is a case-control study and included only 4 controls for every case, which limits the possibilities to achieve the desired matching.

With access to ICD10 diagnostic codes for both hyponatremia and SIADH, subtyping hyponatremia into subtypes of SIADH and non-SIADH should be possible, at least in theory. Unfortunately, the diagnostic code for SIADH is most likely a poor estimate of this condition, due to insufficient laboratory work-up of most hyponatremic patients (39,40), resulting in poor validity for this diagnostic code.

The current study is a good example of the importance of postmarketing surveillance to reveal hitherto not well-recognized properties of common drugs and to evaluate the real-world effectiveness and safety of the drug (41,42). This is in contrast to many randomized controlled trials where patients initiated on antihypertensive treatment other that diuretics have not shown severe hyponatremia. This is likely due to the low absolute risk; randomized controlled trials are usually underpowered to detect severe hyponatremia, sodium levels are seldom reported, and elderly with multiple comorbidities are normally excluded. Unfortunately, due to the design of the current study we did not have the ability to accurately assess absolute risk. However, it should be noted that even highly significant aORs, as seen in this study, might still be associated with quite low absolute risk for individual patients. Although the results need to be confirmed in other settings using different study designs, the findings of this study may have important clinical implications. For a patient with a clinically significant hyponatremia, recent initiation of a vascular CCB, an ACEI, ARB, or BB, should raise suspicion of a causal relationship, and switching from other drug classes to CCBs may not ameliorate this problem. However, for a patient with ongoing treatment, other causes should primarily be sought.

In conclusion, newly initiated treatment with CCBs, BBs, ACEIs, and ARBs was associated with a moderately increased risk for hospitalization due to hyponatremia. In contrast, there was no evidence that ongoing treatment with the investigated antihypertensive drugs increased the risk for hospitalization due to hyponatremia.

## Data Availability

The datasets generated during and/or analyzed during the current study are not publicly available but are available from the corresponding author on reasonable request.

## Additional Information

***Disclosure Summary:*** JS and BM report modest consultancy fees from Otsuka Pharma Scandinavia AB, outside the submitted work. The other authors have nothing to disclose.
